# DIFFERENCES IN BEHAVIOR CAPTURE USING ELECTRONIC MEDICAL RECORD OF NURSING HOMES ACROSS RESIDENT RACE AND ETHNICITY

**DOI:** 10.1093/geroni/igae098.0117

**Published:** 2024-12-31

**Authors:** Hyunkyung Yun, Ellen McCreedy

**Affiliations:** Brown University, Providence, Rhode Island, United States; Veterans’ Administration, Providence, Rhode Island, United States

## Abstract

Agitated behaviors are under-reported in the Minimum Data Set (MDS), a standardized assessment of nursing home (NH) residents, which poses challenges for pragmatic trialists hoping to use routinely collected data to assess study eligibility and outcomes. Our recent study found that using Interventions to Reduce Acute Care Transfers (eINTERACT) and physician orders from EMR increases behavior identification over the MDS alone by 25.7% relative increase (14.8% (SD 35.6) by MDS only vs. 18.6% (SD 38.9) by supplemented by EMR) among NH long-stay residents with dementia. We extended that work to consider variations in behavioral capture by race and ethnicity. We used MDS and EMR from one large, non-profit corporation serving 19,705 residents from 312 NHs between January 2020 and August 2022. We found Black residents were more likely (11.2% vs. 10.5% in total sample) to be in NHs regularly using EMR, defined as the top quartile NHs based on the percent of residents in the NH with eINTERACT form; Latinx residents were less likely to be in NHs regularly using EMR (3.5% vs. 6.0% in total sample). Among NHs regularly using EMR (n=5,192 residents, 81 NHs), Black residents were less likely to have behaviors documented in both MDS (Blacks 9.7% vs. Whites 16.5%) and EMR (eINTERACT: Blacks 1.9% vs. Whites 3.7%; Orders: Blacks 2.6% vs. Whites 3.9%) despite higher share of moderate/severe cognitive impairment (63.1% vs. Whites 60.0%) and less antipsychotic use (16.4% vs. Whites 26.2%). This has implications for inclusion in pragmatic trials and receipt of possibly advantageous treatments.

